# High hepatic insulin resistance is linked to glucose and lipid profiles in Korean adults with type 2 diabetes

**DOI:** 10.3389/fendo.2026.1791174

**Published:** 2026-04-02

**Authors:** Paul Kim, Minji Kang, Hyunji Sang, Sang Youl Rhee, Hyunjung Lim

**Affiliations:** 1 Department of Medical Nutrition, Graduate School of East-West Medical Science, Kyung Hee University, Yongin, Republic of Korea; 2 Research Institute of Medical Nutrition, Kyung Hee University, Seoul, Republic of Korea; 3 Department of Endocrinology and Metabolism, Kyung Hee University Hospital, Kyung Hee University College of Medicine, Seoul, Republic of Korea; 4 Center for Digital Health, Medical Science Research Institute, Kyung Hee University Medical Center, Kyung Hee University College of Medicine, Seoul, Republic of Korea

**Keywords:** cross-sectional, hepatic insulin resistance, HIRI, insulin resistance, Korean, lipid profile, type 2 diabetes mellitus

## Abstract

**Aims:**

To characterize the metabolic profiles associated with the Hepatic Insulin Resistance Index (HIRI), in Korean patients with type 2 diabetes mellitus.

**Methods:**

This cross-sectional study analyzed 2,475 Korean adults with type 2 diabetes from the Korean National Diabetes Program cohort. Participants were categorized into HIRI tertiles based on oral glucose tolerance tests. We examined associations between HIRI and anthropometric measures, glycemic parameters, lipid profiles, liver function markers, and dietary intake. Logistic regression assessed the risk of dyslipidemia and metabolic syndrome components, adjusting for relevant confounders.

**Results:**

High-HIRI participants demonstrated greater central obesity (waist circumference 90.92 vs. 85.52 cm, *p* < 0.0001) and distinctive glycemic profiles: elevated fasting glucose (153.26 vs. 147.53 mg/dL, *p* < 0.0001) but lower postprandial glucose (273.73 vs. 299.32 mg/dL, *p* = 0.0001) and HbA1c (7.8 vs. 8.2%, *p* = 0.0026). They exhibited dyslipidemia with higher triglycerides (190.71 vs. 141.41 mg/dL, *p* < 0.0001) and lower HDL cholesterol (45.29 vs. 48.40 mg/dL, *p* < 0.0001).

**Conclusions:**

High hepatic insulin resistance in Korean patients with type 2 diabetes defines a phenotype characterized by central obesity, atherogenic dyslipidemia, and distinctive glycemic profiles. Recognition of this phenotype supports personalized diabetes management targeting hepatic IR.

## 1 Introduction

Insulin resistance (IR) is a core pathophysiological mechanism in type 2 diabetes (T2D) and a key independent risk factor for cardiovascular disease (1, 2). Recent studies have demonstrated that while IR is a systemic phenomenon, it exhibits distinct metabolic characteristics in major target organs including the liver (hepatic), skeletal muscle, and adipose tissue (3).

The relative contribution of IR in each organ results in distinct clinical manifestations (4). For instance, skeletal muscle IR predominantly impairs postprandial glucose disposal, whereas hepatic IR is a primary determinant of fasting hyperglycemia and directly promotes atherogenic dyslipidemia (5). This distinction is clinically crucial because it suggests that a patient’s specific metabolic risk profile—whether dominated by hyperglycemia or dyslipidemia—may depend on the primary site of IR (6). This provides a strong rationale for investigating organ-specific IR beyond systemic measures.

Among these organ-specific resistances, hepatic IR is distinguished from that in skeletal muscle and adipose tissue by its unique pathophysiological characteristics (4). A key phenomenon observed in the liver is “selective hepatic insulin resistance,” a paradoxical state where insulin fails to suppress gluconeogenesis, yet its signaling for *de novo* lipogenesis is preserved or even enhanced (7). In contrast, skeletal muscle IR is primarily characterized by impaired glucose uptake, while adipose tissue IR manifests as a failure to suppress lipolysis. Therefore, hepatic IR, with its dual impact on glucose and lipid metabolism, simultaneously drives hyperglycemia and dyslipidemia through VLDL overproduction (5).

This dual metabolic burden is particularly pronounced in East Asian populations, who are predisposed to ectopic fat accumulation even at a lower body mass index (8). Consequently, hepatic IR, primarily driven by Metabolic dysfunction-Associated Steatotic Liver Disease (MASLD), represents a key metabolic phenotype in this demographic (9). Furthermore, hepatic fat accumulation is not merely a localized issue; it is a central driver of systemic IR, with strong links to skeletal muscle IR (10).

Therefore, this study aimed to characterize the distinct glucose and lipid metabolic profiles associated with hepatic IR, and to explore their association with cardiovascular risk markers, in Korean patients with T2D. Through this characterization, we sought to provide deeper insights into the underlying pathophysiology and help identify distinct metabolic phenotypes for targeted therapeutic strategies.

## 2 Methods

### 2.1 Data source

This cross-sectional study analyzed data from the Korea National Diabetes Program (KNDP) cohort study. KNDP is a prospective, multicenter observational study initiated in 2006 to enhance clinical and pathophysiological understanding of Korean patients with type 2 diabetes and individuals at high risk for diabetes. The detailed design of the KNDP cohort has been reported previously (11). The observation period was defined from baseline to March 2014. Eligible participants were patients with T2D aged over 19 years. The exclusion criteria were as follows: patients who had no oral glucose tolerance test (OGTT) records and those who could not be analyzed because of data entry errors. A total of 2,475 patients remained for analysis after exclusion criteria were implemented. Individual informed consent was exempted due to the retrospective nature of the data collection. The study protocol was approved by the institutional review boards of the Kyung Hee University Hospital (KMC IRB 0526-04).

### 2.2 Clinical and laboratory measurements

Clinical and laboratory data were extracted from KNDP at the time of patient visits. This included data on diabetes medication use, smoking status, and alcohol consumption. Smoking status was classified as never-smoker (no history of smoking), ever-smoker (≥10 packs of cigarettes over a lifetime), or current smoker (ever-smoker who currently smokes). Alcohol consumption status was classified as never-drinker (no history of drinking), ever-drinker (history of consuming ≥1–2 glasses of beer), or current drinker (ever-drinker who currently consumes alcohol). Physical measurements encompassed height, weight, body mass index (BMI), systolic blood pressure (SBP), and diastolic blood pressure (DBP). BMI was determined using the standard calculation: weight (kg) divided by height squared (m²).

Glucose metabolism markers were obtained through an OGTT. The glucose metabolism parameters analyzed included HbA1c, fasting plasma glucose, and 2-hour postprandial blood glucose. To assess pancreatic beta-cell function and whole-body IR, the Insulinogenic Index (IGI) and the Homeostatic Model Assessment for Insulin Resistance (HOMA-IR) were calculated. The IGI was computed as the ratio of the incremental change in insulin to glucose during the first 30 minutes of the OGTT: (ΔInsulin [0–30 min]/ΔGlucose [0–30 min]) (12). HOMA-IR was calculated using the formula: [fasting insulin (μU/mL) × fasting glucose (mmol/L)]/22.5 (13). Additional laboratory parameters encompassed the lipid profile (total cholesterol, triglycerides, LDL cholesterol, and HDL cholesterol), hepatic function tests (AST, ALT, and gamma-glutamyl transferase), and kidney function indicators (blood urea nitrogen, serum creatinine, estimated creatinine clearance, and urine microalbumin). Estimated creatinine clearance was computed using the MDRD equation: 186 × creatinine^(-1.154)^ × age^(-0.203)^ (× 0.742 for females) (14).

### 2.3 Dietary assessment

Dietary intake was assessed using 3-day food records collected from KNDP during their hospital visits to evaluate participants’ usual dietary patterns. The assessment included intake of energy, macronutrients (carbohydrates, protein, and fat), and minerals such as calcium. Dietary analysis was performed using CAN-Pro 5.0 (Korean Nutrition Society, Republic of Korea).

### 2.4 Definition of dyslipidemia and cardiometabolic risk factors

Dyslipidemia was defined as the presence of one or more of the following: high total cholesterol (≥180 mg/dL), high triglycerides (≥150 mg/dL), low HDL cholesterol (<40 mg/dL for men, <50 mg/dL for women), or high LDL cholesterol (≥100 mg/dL), or use of lipid-lowering medication (15). And components of metabolic syndrome were defined based on the Harmonized National Cholesterol Education Program Adult Treatment Panel III (NCEP ATP III) criteria (16). However, the waist circumference (WC) criteria were adapted for Korean adults, defining high WC as ≥ 90 cm for men and ≥ 85 cm for women (17). Hypertension was defined as a systolic blood pressure ≥130 mmHg, a diastolic blood pressure ≥85 mmHg, or use of antihypertensive medication. Metabolic syndrome was diagnosed if three or more of these risk factors were present.

### 2.5 Hepatic insulin resistance index

The hepatic-specific IR index was derived from the OGTT. The Hepatic Insulin Resistance Index (HIRI), which has been validated against the gold standard insulin clamp technique, was calculated by multiplying the area under the curve (AUC) for plasma glucose and plasma insulin concentrations from 0 to 30 minutes during the OGTT (18). This index has been utilized in previous studies and has demonstrated its clinical utility in assessing hepatic insulin sensitivity (19–21). To account for sex differences in hepatic IR, participants were first stratified by sex, and then categorized into tertiles based on their HIRI values within each sex group, following the approach used in previous research (20, 22).

### 2.6 Statistical analysis

All variables were expressed as mean ± standard deviation for continuous variables or as frequency (%) for categorical variables. Normality testing was performed for all continuous variables, and group differences were assessed using analysis of variance (ANOVA) or Kruskal-Wallis test depending on the distribution of data. When significant differences were identified, *post-hoc* analysis was conducted using Bonferroni correction for multiple comparisons. Spearman’s rank correlation analysis was used to assess the associations between variables. Logistic regression analysis was performed to examine the incidence of dyslipidemia, metabolic syndrome, and its individual components. The regression models were adjusted for covariates including sex, age, BMI, duration of diabetes, smoking status, and alcohol consumption. A *p*-value of less than 0.05 was considered statistically significant. All statistical analyses were conducted using SAS version 9.4 (SAS Institute Inc., Cary, NC, USA).

## 3 Results

### 3.1 Study population characteristics

**Figure 1:**
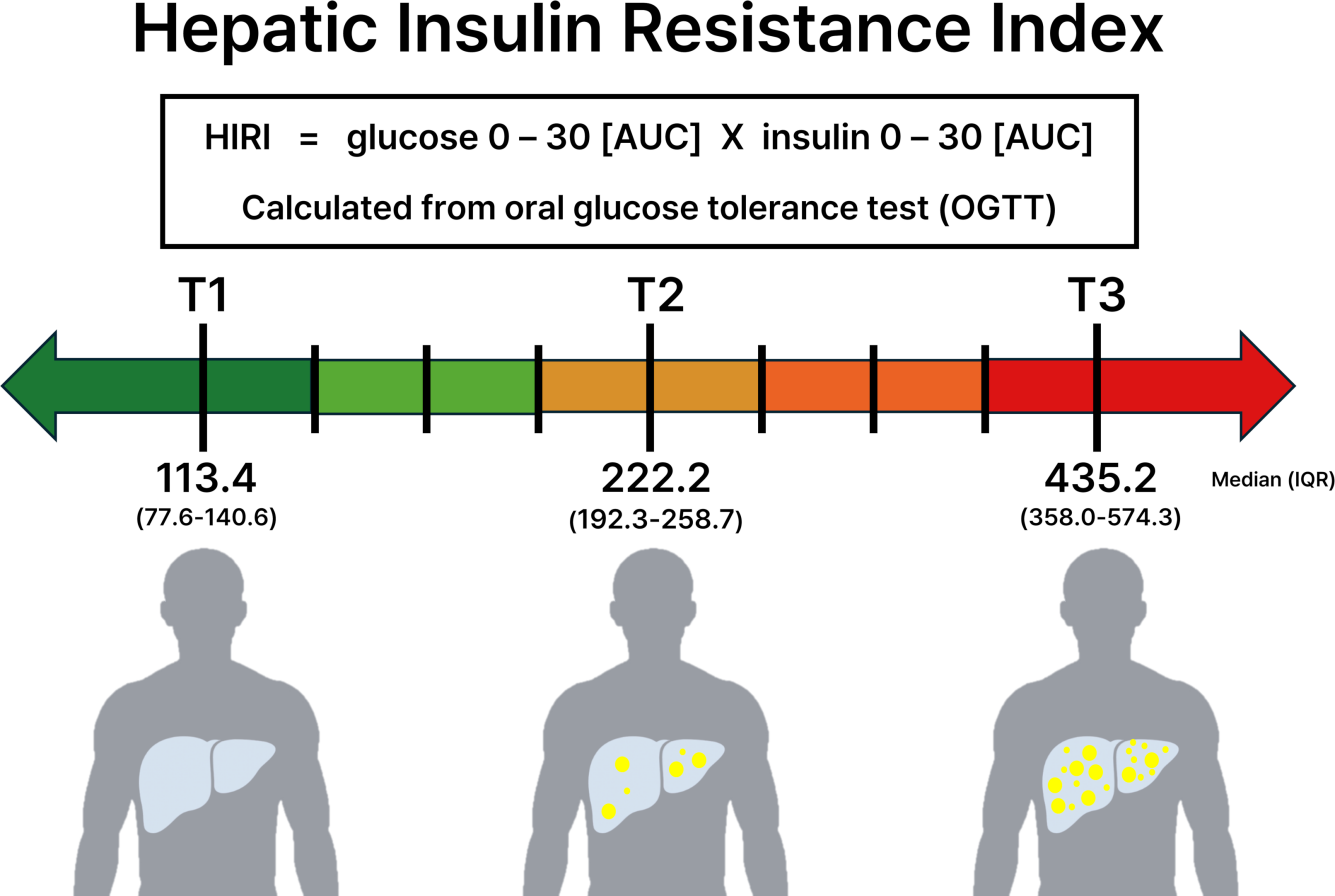
Tertiles of the Hepatic Insulin Resistance Index (HIRI) in Korean adults with type 2 diabetes. HIRI was calculated as glucose AUC_0–30_ × insulin AUC_0–30_ during the OGTT. Participants were categorized into HIRI tertiles defined separately in men and women and then combined into T1–T3 groups (T1, lowest; T3, highest). Values are presented as median (interquartile range). The horizontal color gradient (green to red) indicates increasing HIRI.

A total of 2,475 Korean patients with type 2 diabetes mellitus were included in the analysis, comprising 1,393 (56%) men and 1,082 (44%) women. Participants were divided into tertiles based on HIRI values: low-HIRI (n = 824), middle-HIRI (n = 826), and high-HIRI (n = 825) groups. The median HIRI values were 113.38 (IQR: 77.58-140.63), 222.16 (IQR: 192.32-258.74), and 435.17 (IQR: 358.02-574.32) for the low, middle, and high tertiles, respectively (**Table 1**) (**Figure 1**).

**Table 1:** Anthropometric, lifestyle, and clinical profiles by hepatic insulin resistance index (HIRI) tertiles in Korean type 2 diabetes.

Variables^1)^	Low-HIRI (n = 824)	Middle-HIRI (n = 826)	High-HIRI (n = 825)	*P*-value^†^	*P* for trend^††^
Age (year)	52.91 ± 10.49	53.62 ± 10.31	53.40 ± 10.57	0.3557	0.2806
Height (cm)	162.34 ± 8.79	162.87 ± 8.58	162.85 ± 9.13	0.4900	0.3220
Weight (kg)	63.41 ± 9.97^c^	66.77 ± 10.87^b^	70.74 ± 11.82	**<.0001**	<.0001
BMI (kg/m^2^)	24.00 ± 2.90^c^	25.10 ± 3.07^b^	26.58 ± 3.25^a^	**<.0001**	<.0001
Hip circumference (cm)	94.54 ± 6.43^c^	96.35 ± 6.55^b^	98.18 ± 6.57^a^	**<.0001**	<.0001
Waist circumference (cm)	85.52 ± 7.87^c^	87.94 ± 7.96^b^	90.92 ± 8.27^a^	**<.0001**	<.0001
Systolic blood pressure (mmHg)	124.95 ± 15.23	125.93 ± 15.35	126.70 ± 15.13	0.0557	0.0165
Diastolic blood pressure (mmHg)	78.65 ± 9.51^b^	79.29 ± 9.94^ab^	80.75 ± 9.83^a^	**0.0232**	0.0062
Smoking					
Never-smokers	431 (54)	433 (53)	432 (53)		
Ex-smokers	187 (23)	211 (21)	216 (27)	0.4395	0.6327
Current smokers	187 (23)	168 (26)	167 (21)		
Alcohol consumption status					
Never-drinkers	327 (41)	348 (43)	354 (43)		
Ex-drinkers	108 (13)	92 (11)	90 (11)	0.5378	0.2586
Current drinkers	369 (46)	372 (46)	372 (46)		
Diabetes duration (year)	4.87 ± 6.04^a^	4.80 ± 6.19^a^	3.58 ± 4.94^b^	**0.0004**	0.0002

BMI, body mass index (Weight(kg)/Height(m)^2^).

^1)^Values are expressed as Mean ± Standard Deviation as continuous variables and Number (%) as categorial variables.

^†^*P*-values calculated using ANOVA or Kruskal–Wallis test, depending on data normality.

^††^*P* for trends were obtained by the Jonckheere-Terpstra test for continuous variables, Cochran-Mantel-Haenszel statistics for categorical variables.

Values with different superscript letters are significantly different (*p*  < 0.05) by Bonferroni *post hoc* test. Bold-faced p-values indicate statistical significance (*p*-value < 0.05).

Participants with higher HIRI demonstrated significantly elevated anthropometric measurements compared to those with lower HIRI. While height showed no significant difference across tertiles (*p* = 0.4900), body weight increased from the low-HIRI to high-HIRI (63.41 ± 9.97 kg vs. 70.74 ± 11.82 kg, *p* < 0.0001), resulting in correspondingly higher BMI (24.00 ± 2.90 kg/m² vs. 26.58 ± 3.25 kg/m², *p* < 0.0001). Both hip (94.54 ± 6.43 cm vs. 98.18 ± 6.57 cm, *p* < 0.0001) and WC (85.52 ± 7.87 cm vs. 90.92 ± 8.27 cm, *p* < 0.0001) showed positive correlations with HIRI. Additionally, both systolic and diastolic blood pressure exhibited increasing trends across tertiles, with diastolic blood pressure showing statistical significance (78.65 ± 9.51 mmHg vs. 80.75 ± 9.83 mmHg, *p* = 0.0232). Notably, diabetes duration was significantly shorter in the high-HIRI group compared to the low-HIRI group (3.58 ± 4.94 years vs. 4.87 ± 6.04 years, *p* = 0.0004), showing a decreasing trend across tertiles (*p* for trend=0.0002).

### 3.2 Correlations between HIRI and clinical parameters

**Figure 2:**
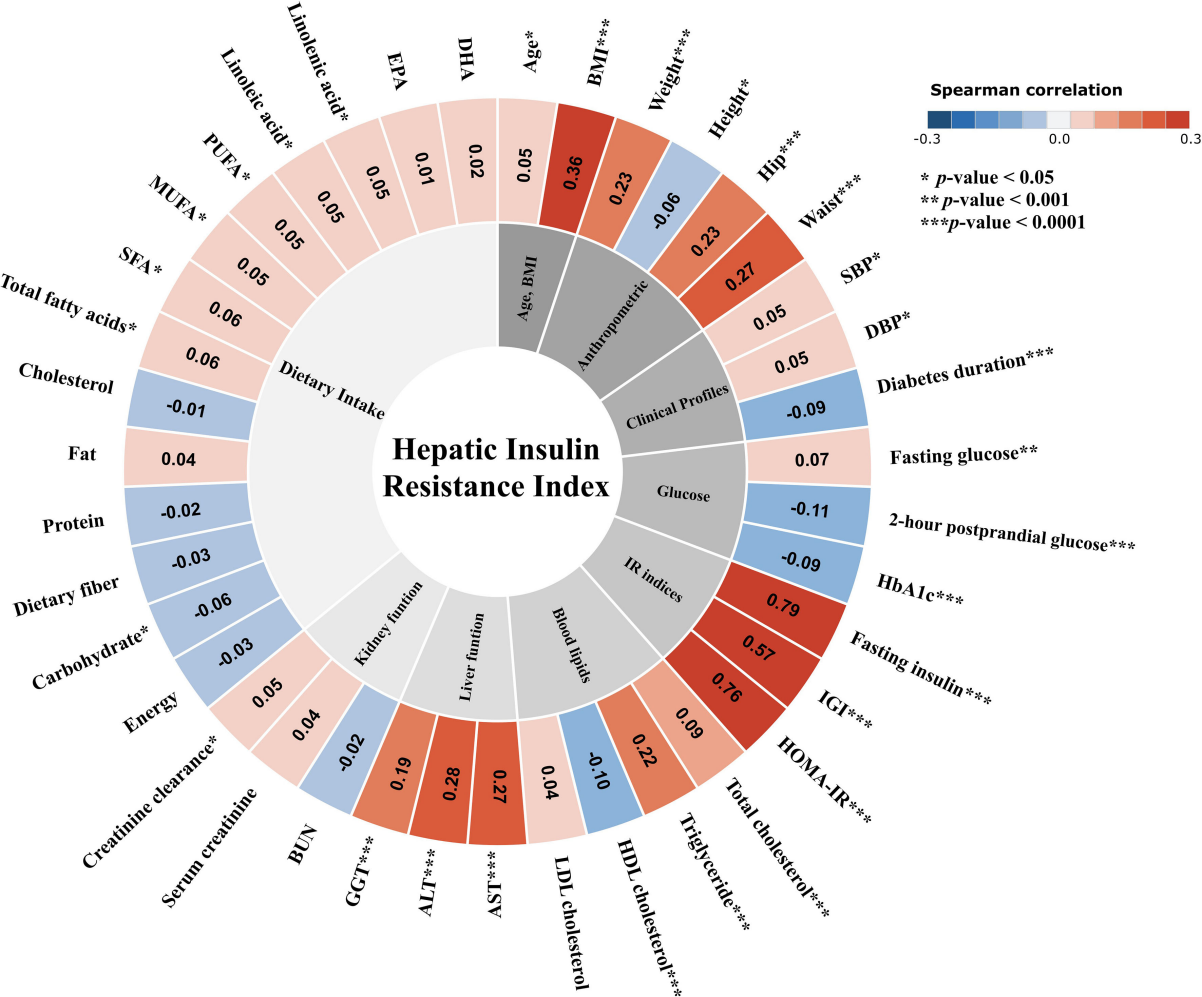
Spearman correlation map between the Hepatic Insulin Resistance Index (HIRI) and metabolic variables. Variables are grouped by major domains (inner ring): age and BMI, anthropometric measures, clinical profiles, glucose parameters, insulin resistance (IR) indices, blood lipids, liver function, kidney function, and dietary intake. Each segment displays Spearman’s correlation coefficient (ρ) between HIRI and the corresponding variable, and the color scale denotes the direction and magnitude of the correlation (blue, negative; red, positive; range −0.3 to 0.3). Statistical significance is indicated by asterisks (^*^p < 0.05, ^**^p < 0.001, ^***^p < 0.0001).

The correlations between HIRI and various clinical parameters are summarized in **Figure 2**. HIRI showed positive correlations with anthropometric measures including BMI and WC. For glycemic parameters, HIRI demonstrated a positive correlation with fasting glucose while showing negative correlations with 2-hour postprandial glucose and HbA1c. Regarding lipid profiles, HIRI was positively correlated with total cholesterol and triglycerides and negatively correlated with HDL cholesterol. HIRI also showed positive correlations with liver function markers, including ALT, AST, and GGT. By contrast, correlations with dietary intake were generally weak or non-significant, although modest associations were observed for certain fatty acid subtypes and carbohydrate intake.

### 3.3 Metabolic profiles associated with HIRI

Beyond correlation analysis, we compared metabolic characteristics across HIRI tertiles (**Table 2**). The high-HIRI group had significantly higher fasting glucose levels compared to the low-HIRI group (153.26 ± 52.49 mg/dL vs. 147.53 ± 59.65 mg/dL), with significant differences observed among the three groups (*p* < 0.0001). Interestingly, both 2-hour postprandial glucose (273.73 ± 99.55 mg/dL vs. 299.32 ± 110.09 mg/dL; *p* = 0.0001) and HbA1c (7.79 ± 1.88% vs. 8.18 ± 2.18%; *p* = 0.0026) were significantly lower in the high-HIRI group. Indices of IR and secretion were markedly higher with increasing HIRI, including fasting insulin, HOMA-IR, and IGI (*p* < 0.0001). HOMA-IR, for example, was approximately three times higher in the high-HIRI than in the low-HIRI (5.45 ± 4.94 vs. 1.37 ± 0.81).

**Table 2:** Hepatic insulin resistance index (HIRI) and associated metabolic profiles in Korean patients with type 2 diabetes.

Variables^1)^	Low-HIRI (n = 824)	Middle-HIRI (n = 826)	High-HIRI (n = 825)	*P*-value^†^
Glucose				
Fasting glucose (mg/dL)	147.53 ± 59.65^b^	151.48 ± 51.37^a^	153.26 ± 52.49^a^	**<.0001**
2-hour postprandial glucose (mg/dL)	299.32 ± 110.09^a^	290.82 ± 102.63^a^	273.73 ± 99.55^b^	**0.0001**
HbA1c (%)	8.2 ± 2.9^a^	8.0 ± 1.9^ab^	7.8 ± 1.9^b^	**0.0026**
Insulin resistance indices				
Fasting insulin (μU/mL)	3.92 ± 2.27^c^	7.62 ± 3.25^b^	14.23 ± 12.92^a^	**<.0001**
Insulinogenic index	0.06 ± 0.08^c^	0.12 ± 0.12^b^	0.31 ± 0.45^a^	**<.0001**
HOMA-IR	1.37 ± 0.81^c^	2.79 ± 1.23^b^	5.45 ± 4.94^a^	**<.0001**
Blood lipids				
Total cholesterol (mg/dL)	184.11 ± 39.24^b^	190.06 ± 44.57^a,b^	191.63 ± 41.39^a^	**0.0018**
Triglyceride (mg/dL)	141.41 ± 90.80^c^	177.09 ± 145.01^b^	190.71 ± 138.06^a^	**<.0001**
HDL cholesterol (mg/dL)	48.40 ± 12.61^a^	47.18 ± 12.14^a^	45.29 ± 11.63^b^	**<.0001**
LDL cholesterol (mg/dL)	107.95 ± 35.46	109.47 ± 38.85	110.45 ± 36.51	0.4244
Liver function				
AST (U/L)	22.87 ± 18.21^c^	25.68 ± 14.17^b^	29.54 ± 18.20^a^	**<.0001**
ALT (U/L)	25.22 ± 24.59^c^	29.84 ± 20.89^b^	37.24 ± 27.48^a^	**<.0001**
Gamma-glutamyl transferase (IU/L)	40.17 ± 52.72^c^	52.01 ± 83.89^b^	53.96 ± 68.15^a^	**<.0001**
Kidney function				
BUN (mg/dL)	14.74 ± 4.71	14.51 ± 4.46	14.50 ± 4.45	0.8992
Serum creatinine (mg/dL)	0.78 ± 0.22^b^	0.82 ± 0.22^a^	0.83 ± 0.24^a^	**<.0001**
Creatinine clearance (ml/min)	97.62 ± 31.11^b^	96.81 ± 29.89^b^	102.71 ± 35.58^a^	**0.0023**

HbA1c: Hemoglobin A1c; HOMA-IR: Homeostatic Model Assessment for Insulin Resistance; HDL: High-Density Lipoprotein; LDL: Low-Density lipoprotein; AST: Aspartate Transaminase; ALT: Alanine Transaminase; BUN: Blood Urea Nitrogen.

^1)^Values are expressed as Mean ± Standard Deviation as continuous variables.

^†^*P*-values calculated using ANOVA or Kruskal–Wallis test, depending on data normality.

Values with different superscript letters are significantly different (*p* < 0.05) by Bonferroni *post hoc* test. Bold-faced p-values indicate statistical significance (*p*-value < 0.05).

The lipid profile also showed significant associations with HIRI. The high-HIRI demonstrated significantly higher levels of total cholesterol (191.63 ± 41.39 mg/dL vs. 184.11 ± 39.24 mg/dL; *p* = 0.0018) and triglycerides (190.71 ± 138.06 mg/dL vs. 141.41 ± 90.80 mg/dL; *p* < 0.0001). In contrast, HDL cholesterol levels were significantly lower in the high-HIRI (45.29 ± 11.63 mg/dL vs. 48.40 ± 12.61 mg/dL; *p* < 0.0001). There was no significant difference in LDL cholesterol levels among the three groups (*p* = 0.4244). Liver function markers, including AST, ALT, and Gamma-glutamyl Transferase, were all significantly higher in the high-HIRI compared to the low-HIRI (*p* < 0.0001).

Regarding kidney function, BUN levels did not differ significantly among the groups (*p* = 0.8992). However, both serum creatinine (0.83 ± 0.24 mg/dL vs. 0.78 ± 0.22 mg/dL; *p* < 0.0001) and creatinine clearance (102.71 ± 35.58 mL/min vs. 97.62 ± 31.11 mL/min; *p* = 0.0023) were significantly higher in the high-HIRI compared to the low-HIRI.

### 3.4 Dietary nutrient intake

Dietary analyses were conducted using available dietary data across the HIRI tertiles (**Table 3**). There were no significant differences in total daily energy intake (*p* = 0.3322), total carbohydrate intake (*p* = 0.4190), or total protein intake (*p* = 0.1367) among the groups. However, when analyzed as a proportion of total energy or relative to body weight, several differences emerged.

**Table 3:** Dietary intake according to hepatic insulin resistance index (HIRI) tertile groups in Korean patients with type 2 diabetes.

Variables^1)^	Low-HIRI (n = 610)	Middle-HIRI (n = 604)	High-HIRI (n = 622)	*P*-value^†^
Energy (kcal)	1791.58 ± 420.58	1788.88 ± 452.58	1813.42 ± 413.17	0.3322
Carbohydrate (g)				
Energy from carbohydrates (%)	61.01 ± 9.52^a^	60.8 ± 9.52^a^	59.54 ± 9.57^b^	**0.0078**
Total carbohydrates (g)	269.77 ± 62.40	267.62 ± 60.84	266.29 ± 60.99	0.4190
Dietary fiber (g)	27.39 ± 9.37	26.80 ± 9.13	26.67 ± 8.83	0.4066
Protein (g)				
Energy from protein (%)	17.27 ± 3.39	16.94 ± 3.13	17.21 ± 3.24	0.1773
Total protein (g)	77.46 ± 23.65	75.63 ± 22.91	77.99 ± 22.64	0.1367
Protein (g/kg body weight)	1.25 ± 0.41^a^	1.16 ± 0.36^b^	1.12 ± 0.35^b^	**<.0001**
Fat (g)				
Energy from fat (%)	21.53 ± 7.98^b^	21.61 ± 7.43^ab^	22.49 ± 7.41^a^	**0.0062**
Total fat (g)	43.97 ± 23.19^b^	44.19 ± 22.62^ab^	46.31 ± 21.11^a^	**0.0117**
Cholesterol (mg)	267.01 ± 184.70	251.42 ± 183.13	273.02 ± 189.58	0.0697
Total fatty acids (g)	23.48 ± 17.86^b^	24.72 ± 20.78^b^	26.58 ± 18.80^a^	**0.0008**
Saturated fatty acids (g)	6.81 ± 6.71^b^	7.55 ± 8.35^ab^	7.89 ± 7.25^a^	**0.0022**
Monounsaturated fatty acid (g)	8.61 ± 7.60^b^	9.15 ± 9.11^b^	9.83 ± 8.20^a^	**0.0019**
Polyunsaturated fatty acid (g)	8.06 ± 5.32^b^	8.01 ± 5.03^b^	8.87 ± 5.33^a^	**0.0017**
Linoleic acid (g)	6.59 ± 4.53^b^	6.73 ± 4.55^ab^	7.28 ± 4.64^a^	**0.0082**
Linolenic acid (g)	0.65 ± 0.53^b^	0.64 ± 0.47^b^	0.73 ± 0.53^a^	**0.0024**
Eicosapentaenoic acid (g)	0.16 ± 0.39	0.12 ± 0.32	0.17 ± 0.40	0.0636
Docosahexaenoic acid (g)	0.36 ± 0.80^ab^	0.27 ± 0.69^b^	0.39 ± 0.82^a^	**0.0223**

^1)^Values are expressed as Mean ± Standard Deviation as continuous variables.

^†^*P*-values calculated using ANOVA or Kruskal–Wallis test, depending on data normality.

Values with different superscript letters are significantly different (*p* < 0.05) by Bonferroni *post hoc* test. Bold-faced p-values indicate statistical significance (*p*-value < 0.05).

The percentage of energy from carbohydrates was significantly lower in the high-HIRI compared to the low-HIRI (59.54 ± 9.57% vs. 61.01 ± 9.52%; *p* = 0.0078). In contrast, the percentage of energy from fat was significantly higher (22.49 ± 7.41% vs. 21.53 ± 7.98%; *p* = 0.0062). When adjusted for body weight, protein intake (1.12 ± 0.35 g/kg BW vs. 1.25 ± 0.41 g/kg BW; *p* < 0.0001) was significantly lower in the high-HIRI.

An analysis of fat composition revealed that the high-HIRI group consumed a significantly greater amount of total fat (46.31 ± 21.11 g vs. 43.97 ± 23.19 g; *p* = 0.0117). This was reflected across various fatty acid types, with higher intake of total fatty acids (*p* = 0.0008), saturated fatty acids (*p* = 0.0022), monounsaturated fatty acids (*p* = 0.0019), and polyunsaturated fatty acids (*p* = 0.0017) in the high-HIRI. Intake of dietary fiber, and cholesterol did not differ significantly across the tertiles.

### 3.5 Association of HIRI with dyslipidemia and cardiometabolic risk factors

To examine the association between HIRI and various cardiometabolic risk factors, we performed logistic regression analyses using the low-HIRI group as the reference (**Table 4**). Two models were constructed: an unadjusted model and a multivariate model adjusted for age, sex, BMI, smoking, drinking status and duration of diabetes.

**Table 4:** Odds ratios for cardiometabolic risk factors according to hepatic insulin resistance index (HIRI) tertile groups in Korean patients with type 2 diabetes.

Outcome	Middle-HIRI	High-HIRI	*P* for trend^†^
	Odd Ratio (95% CI)	Odd Ratio (95% CI)	
High waist circumference			
Unadjusted	1.597 (1.311 - 1.945)	2.616 (2.138 - 3.201)	**<.0001**
Adjusted^1)^	0.962 (0.733 - 1.262)	0.879 (0.657 - 1.175)	0.3871
Hypertension			
Unadjusted	1.254 (1.023 - 1.536)	1.753 (1.434 - 2.143)	**<.0001**
Adjusted^1)^	1.068 (0.854 - 1.335)	1.312 (1.040 - 1.656)	**0.0217**
High fasting glucose			
Unadjusted	2.034 (1.459 - 2.835)	1.965 (1.409 - 2.740)	**<.0001**
Adjusted^1)^	1.953 (1.375 - 2.774)	1.863 (1.283 - 2.705)	**0.0004**
High triglyceride			
Unadjusted	1.679 (1.367 - 2.062)	2.241 (1.824 - 2.754)	**<.0001**
Adjusted^1)^	1.483 (1.187 - 1.854)	1.669 (1.322 - 2.107)	**<.0001**
Low HDL cholesterol			
Unadjusted	1.177 (0.957 - 1.447)	1.531 (1.247 - 1.881)	**<.0001**
Adjusted^1)^	1.146 (0.913 - 1.439)	1.352 (1.066 - 1.715)	**0.0129**
High LDL cholesterol			
Unadjusted	0.954 (0.776 - 1.172)	1.063 (0.865 - 1.307)	0.5660
Adjusted^1)^	0.927 (0.744 - 1.155)	0.930 (0.738 - 1.173)	0.5366
High total cholesterol			
Unadjusted	1.049 (0.854 - 1.288)	1.306 (1.066 - 1.599)	**0.0096**
Adjusted^1)^	1.041 (0.840 - 1.290)	1.201 (0.969 - 1.489)	0.0942
Dyslipidemia^2)^			
Unadjusted	1.482 (1.193 - 1.841)	1.676 (1.350 - 2.080)	**<.0001**
Adjusted^1)^	1.413 (1.124 - 1.777)	1.484 (1.167 - 1.887)	**0.0014**
Metabolic syndrome^2)^			
Unadjusted	1.962 (1.559 - 2.469)	2.707 (2.140 - 3.424)	**<.0001**
Adjusted^1)^	1.523 (1.170 - 1.983)	1.435 (1.085 - 1.898)	**0.0014**

^1)^Adjusted for age, sex, BMI, smoking, drinking, and duration of Type 2 Diabetes.

^2)^Definitions based on NCEP ATP III criteria; Hypertension as SBP/DBP ≥130/85 mmHg or use of antihypertensive drugs. Dyslipidemia was defined as having one or more lipid abnormalities or use of lipid-lowering drugs. Metabolic syndrome was defined as having three or more components.

Reference group is Low-HIRI.

^†^*P* for trends were obtained by the Cochran-Armitage Trend Test. Bold-faced p-values indicate statistical significance (*p*-value < 0.05).

The analysis of dyslipidemia components revealed a distinct pattern characteristic of IR. While no significant association was found for high LDL cholesterol, the odds for having low HDL cholesterol and high triglycerides showed an increase with higher HIRI tertiles in the adjusted model. Participants in the high-HIRI showed a higher prevalence of low HDL-cholesterol (adjusted OR, 1.352; 95% CI, 1.066–1.715) and high triglycerides (adjusted OR, 1.669; 95% CI, 1.322–2.107) compared with those in the low-HIRI group. Consequently, the overall prevalence of dyslipidemia was significantly higher in the middle-HIRI and high-HIRI groups, with the latter showing an adjusted OR of 1.484 (95% CI, 1.167–1.887).

This pattern of increased risk extended to other cardiometabolic components. While the crude association showed higher odds for elevated WC in the high-HIRI group, this relationship lost statistical significance after adjustment. In contrast, hypertension remained significantly associated with the high-HIRI (adjusted OR: 1.312; 95% CI, 1.040–1.656). Consistent with these findings, the prevalence of metabolic syndrome was significantly higher in the high-HIRI group compared to the low-HIRI group. In the adjusted model, the high-HIRI group had significantly increased odds of metabolic syndrome compared to the low-HIRI group (OR, 1.435; 95% CI, 1.085–1.898).

## 4 Discussion

In this study of 2,475 Korean patients with type 2 diabetes, HIRI was associated with a specific and divergent metabolic profile. Specifically, while HIRI showed a strong positive association with fasting plasma glucose, it displayed an inverse relationship with both postprandial 2-hour glucose and HbA1c. This glycemic pattern was accompanied by a pro-atherogenic lipid profile, characterized by higher total cholesterol and triglycerides, and lower HDL-cholesterol.

The HIRI values identified in our study were compared with studies from other countries and ethnic groups, as to our knowledge, no previous studies have examined HIRI in Korea. The PERSON study reported a median HIRI of 601 (IQR: 467-716) in adults with liver IR (20), while the CORDIOPREV-DIAB study reported a mean HIRI of 710 ± 419 in a similar subgroup (23). The MARCH study, which examined hepatic insulin resistance in Chinese patients with diabetes, reported a high-HIRI group range of 299 to 977, which is similar to our high-HIRI group (24).

Higher HIRI was strongly associated with higher BMI and WC across HIRI tertiles in the study population. This is consistent with previous studies in which individuals with liver IR had significantly higher BMI and WC compared to those without IR (23). This finding aligns with the well-established ‘portal theory,’ which posits that free fatty acids and pro-inflammatory factors released from visceral fat are delivered directly to the liver via the portal circulation, thereby exacerbating hepatic insulin resistance (25). Crucially, this accumulation of hepatic fat is the lynchpin that connects central adiposity to the glycemic profile we observed. The buildup of lipotoxic intermediates (e.g., diacylglycerols) within hepatocytes is known to selectively impair insulin’s ability to suppress gluconeogenesis, thus leading to elevated fasting glucose (10).

Simultaneously, this severe hepatic IR state triggers compensatory hyperinsulinemia from pancreatic β-cells, explaining the paradoxically low postprandial glucose levels in our high-HIRI group. This phenomenon characterizes the “hypercompensatory” phase of insulin resistance-driven diabetes, where β-cells hypersecrete insulin to overcome resistance (26). The PERSON study, comparing muscle IR and hepatic IR, demonstrated that individuals with hepatic IR had higher fasting glucose and insulin levels alongside lower 2-hour postprandial glucose (20). The preserved postprandial glucose handling despite severe hepatic IR suggests that compensatory hyperinsulinemia may partially compensate for peripheral insulin resistance during glucose loading, while the liver’s impaired ability to suppress gluconeogenesis continues to drive fasting hyperglycemia.

Turning to HbA1c, an inverse association with HIRI was observed in the present analysis. A previous study that divided newly diagnosed diabetic patients by HIRI reported that the HbA1c was 8.30% (7.50, 8.90) in the low-HIRI group and 8.20% (7.40, 9.20) in the high-HIRI group, but the difference was not statistically significant (24). Other studies, however, have reported a positive correlation between HIRI and HbA1c (23). These discrepancies across studies warrant consideration of factors that may influence HbA1c when interpreting this association.

There are several possible explanations for these findings. First, the high-HIRI group had a shorter disease duration. While the duration of diabetes may influence HbA1c, it is more important to examine the characteristics of the high-HIRI group. The high-HIRI group itself may represent a distinct phenotype (27). This group tends to be obese and has a higher prevalence of metabolic syndrome and increased risk of dyslipidemia. In diabetic patients, they are more likely to already have other related comorbidities and may have presented with secondary diabetes. This subgroup, which carries a high cardiovascular risk such as atherosclerosis, has likely sustained significant endocrine damage, including to the liver, due to central obesity, which may have contributed to the development of diabetes (28).

Second, the glucose profile of this group is characterized by effective postprandial glucose control, which contributes to lower HbA1c. As is well established, the relative contribution of postprandial glucose to HbA1c is greater in patients with better overall glycemic control. For example, Kang et al. reported that postprandial glucose was the major contributor to HbA1c in newly diagnosed type 2 diabetes patients with HbA1c below 8.5% (29). Finally, elevated liver enzymes were observed in the high-HIRI group. Patients with advanced liver disease such as cirrhosis may have reduced HbA1c reliability due to anemia (30).

This adverse metabolic profile also extended to lipid parameters, as a higher HIRI was strongly associated with the key features of atherogenic dyslipidemia: hypertriglyceridemia and low HDL-cholesterol. This finding is highly consistent with previous studies utilizing hepatic IR, which have robustly demonstrated its association with an adverse lipid profile across diverse populations (31, 32).

This pattern is a classic manifestation of hepatic IR. In this state, the liver fails to suppress gluconeogenesis yet continues to stimulate *de novo* lipogenesis (DNL) due to unopposed SREBP-1c activity (10). This unchecked DNL leads to an overproduction of triglycerides, which are subsequently packaged and secreted as VLDL particles (33). Simultaneously, the resulting triglyceride-rich environment promotes the exchange of triglycerides into HDL particles, making them susceptible to rapid catabolism and thereby lowering circulating HDL-cholesterol levels (34). Together, these alterations create a highly pro-atherogenic lipid profile (35). This atherogenic dyslipidemia has been independently associated with increased carotid intima-media thickness (cIMT), a well-validated non-invasive surrogate marker for subclinical atherosclerosis (36, 37). In obese patients with NAFLD (now termed MASLD), increased cIMT has been reported to correlate with the severity of hepatic steatosis on ultrasonography as well as elevated levels of inflammatory chemokines such as eotaxin (38). Compounding this vascular risk, the central obesity and hepatic lipotoxicity intrinsic to the high-HIRI group are recognized drivers of systemic low-grade inflammation and oxidative stress (39, 40). These processes are known to progressively impair endothelial integrity in large arteries, including the carotid vasculature (40). Collectively, these observations suggest that patients harboring the high-HIRI phenotype may face a substantially elevated risk of subclinical vascular remodeling, extending their clinical burden well beyond hepatic disease progression alone.

Therefore, our findings indicate that the high-HIRI group may not represent a uniform entity but rather a heterogeneous subgroup in which more advanced hepatic pathology could be a principal contributor to their glycemic profile. HIRI may serve as a potential clinical indicator for identifying individuals at higher risk and warrants further evaluation of the underlying etiology of their diabetes, which in some cases may extend beyond typical T2D.

### 4.1 Strengths and limitations

This study has several notable strengths. To our knowledge, this is one of the first studies to comprehensively characterize the metabolic and dietary profiles associated with the HIRI in a large clinical population of Korean patients with type 2 diabetes. Our findings provide valuable pathophysiological insights into a specific phenotype of diabetes prevalent in this population. Another major strength is the comprehensive nature of our data, which encompassed detailed clinical and laboratory parameters in addition to dietary intake information. This multi-faceted approach allowed for a more holistic investigation of the factors associated with hepatic IR. Furthermore, this study was conducted in a real-world clinical population of patients already diagnosed with type 2 diabetes, rather than in healthy or prediabetic individuals, which provides direct clinical relevance for understanding the heterogeneity within the diabetic population itself.

Several limitations of this study must be acknowledged. First, due to the cross-sectional design, causal relationships between HIRI and metabolic abnormalities could not be established. Second, while our study focused on hepatic IR, we acknowledge that IR in other tissues, such as muscle, or other unmeasured factors may act as potential confounders influencing the observed associations.

Given the profound heterogeneity of type 2 diabetes, further research is needed to identify and validate distinct patient subgroups based on their underlying pathophysiology. This stratification will ultimately enable the development of more effective, personalized treatment algorithms that can improve patient outcomes.

## 5 Conclusion

In conclusion, this study highlights that a high HIRI in Korean patients with type 2 diabetes characterizes a distinct, high-risk metabolic phenotype. This phenotype encompasses a cluster of adverse features, including central obesity and atherogenic dyslipidemia, and is further distinguished by a paradoxical glycemic signature of high fasting glucose with low 2-hour postprandial glucose. The HIRI value, therefore, appears to reflect a specific pathophysiological state where severe, liver-centric IR coexists with a robustly compensatory beta-cell response. Acknowledging this profile is an important step towards a more personalized approach to diabetes care, which may involve specifically addressing hepatic IR in this subgroup.

## Data Availability

The data analyzed in this study is subject to the following licenses/restrictions: The data that support the findings of this study are not openly available due to reasons of sensitivity and are available from the corresponding author upon reasonable request. Requests to access these datasets should be directed to Hyunjung Lim.
